# Improving battery safety by reducing the formation of Li dendrites with the use of amorphous silicon polymer anodes

**DOI:** 10.1038/srep13219

**Published:** 2015-08-07

**Authors:** Hitoshi Maruyama, Hideyuki Nakano, Masahiro Ogawa, Masaaki Nakamoto, Toshiaki Ohta, Akira Sekiguchi

**Affiliations:** 1 Department of Chemistry, Graduate School of Pure and Applied Sciences, University of Tsukuba, Tsukuba, Ibaraki 305-8571, Japan; 2 TOYOTA CENTRAL R&D LABS. INC., Nagakute, Aichi 480-1192, Japan; 3 SR Center, Ritsumeikan University, 1-1-1 Noji-Higashi, Kusatsu, Shiga, 525-8577, Japan

## Abstract

To provide safe lithium-ion batteries (LIBs) at low cost, battery materials which lead to reduced Li dendrite formation are needed. The currently used anode materials have low redox voltages that are very close to the redox potential for the formation of Li metal, which leads to severe short circuiting. Herein, we report that when the three-dimensional amorphous silicon polymers poly(methylsilyne) and poly(phenylsilyne) are used as anode materials, dendritic Li formation on the anode surface is avoided up to a practical current density of 10 mA·g^−1^ at 5 °C. Equally as significant, poly(methylsilyne) and poly(phenylsilyne) are capable of reacting with 0.45 and 0.9 Li atoms per formula unit, respectively, at an average voltage of approximately 1.0 V, affording reversible capacities of 244 mAh·g^−1^ and 180 mAh·g^−1^. Moreover, noteworthy is the fact that polysilynes are suitable for practical applications because they can be prepared through a simple and low-cost process and are easy to handle.

Lithium-ion batteries (LIBs) have received increasing attention with the widespread use of portable electronic devices and electric vehicles (EVs)^1^,^2^,^3^. Considering the progress in EVs, the next generation of LIBs will be selected on the basis of their safety, cost and energy capacity^4^. For the cathode material, a concentration-gradient material which exhibits superior performance in thermal-abuse tests^5^ has been proposed. LiFePO_4_^6^ and organic materials^7^ have also been proposed as safer cathode materials. Conversely, while graphite anodes are generally used in LIBs^8^,^9^, silicon^10^,^11^,^12^ and tin^13^,^14^ anodes are promising alternatives because of their higher theoretical capacities. The voltage generated by Li insertion into these anodes, however, is very close to that of Li metal formation^15^. Consequently, when the cells are operated at temperatures below 0 °C or at high density currents, Li metal and/or Li compounds are deposited on the anode surface, causing a short circuit between the electrodes. To increase the redox voltage of the anode system, Li_4_Ti_5_O_12_ has been proposed^16^, which is capable of reacting with one Li species per formula unit at a voltage of 1.5 V, yielding a reversible capacity of 150 mAh·g^−1^. This Li insertion voltage makes Li_4_Ti_5_O_12_ suitable for use in LIBs; however, its capacity is approximately half that of graphite. More recently, conjugated dicarboxylate anodes have been proposed, which yield a reversible capacity of 300 mAh·g^−1^ at a potential of 0.8 V^17^. While these properties make conjugated dicarboxylate suitable for use in LIBs, the questions of how to increase the retention capacity with cycling and how to decrease the first large irreversible capacity still remain.

Our group recently investigated the use of a series of stable radicals centred on persilyl-substituted heavy group-14 elements, (tBu_2_MeSi)_3_E• [E = Si, Ge and Sn], as anode materials^18^. The redox potentials of these compounds are all approximately 1.7 V, leading to smaller energy densities compared with those of conventional anodes. Compared with the recent values reported for bulk silicon, the potentials of organic silicon compounds are considerably higher. Given the sufficiently large selection of organic compounds with low-voltage redox activities and the high likelihood of the large-scale application of EVs, we decided to investigate three-dimensional amorphous polysilyne systems featuring σ-conjugation derived from Si–Si bonds prepared by using eco-efficient materials and processes. The structural features of polysilyne are between those of bulk silicon and organic silicon compounds, and therefore its redox potential should be approximately 1 V (between that of silicon at 0.2 V and that of organic silicon compounds at 1.7 V). Polymeric polysilynes contain Si–Si main chains and organic substituents (R) and can be regarded as compounds in which one of the Si–Si bonds in crystalline silicon has been replaced by a Si–R bond (Fig. 1a).

This polymer structure enables better accommodation of large volume changes without initial fracture. A schematic illustration of the polysilyne anode configuration is shown in Fig. 1b. The theoretical capacities of **1** and **2** are 623 mAh·g^−1^ and 253 mAh·g^−1^, respectively, according to the following redox reaction:


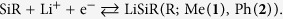


## Results and Discussion

### Structural assignments and half-cell electrochemical characterization

**Figure 1:**
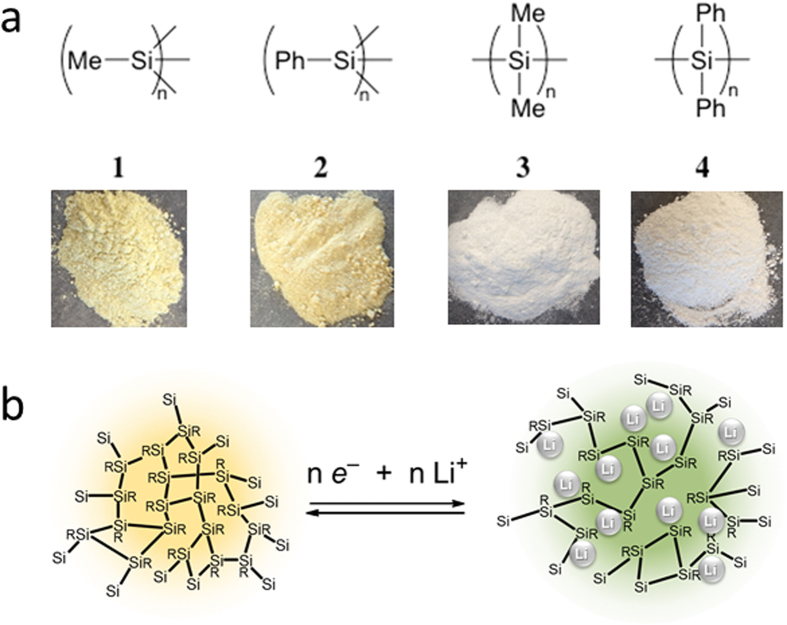
Schematic structures. (**a**) Polysilynes **1** and **2** and polysilanes **3** and **4**. Corresponding photos for each sample are also shown. (**b**) Redox behaviours of polysilynes.

Poly(methylsilyne) (**1**) and poly(phenylsilyne) (**2**) were prepared according to previous reports^19^. For comparative purposes, poly(dimethylsilane) (**3**) and poly(diphenylsilane) (**4**), which have one-dimensional Si–Si chain structures, were also prepared. The obtained polymers were yellow (**1** and **2**) and white (**3** and **4**) powders that were insoluble in organic solvents (Fig. 1a). Scanning electron microscope (SEM) images of the samples revealed featureless particles with sizes ranging from 0.05 μm to 0.5 μm (Fig. S1). Broad signals were observed in the X-ray diffraction (XRD) patterns of **1** and **2** (9.5° (d = 9.3 Å) and 26.5° (d = 3.3 Å), and 8.5° (d = 10.4 Å) and 20.0° (d = 4.4 Å), respectively), indicating that the structures had low crystallinities (Figs S2a and S2b, respectively). Based on this structural information, it was deduced that poly(phenylsilyne) **2** has a cage larger than that of poly(methylsilyne) **1**, which suggests that it also has an increased capacity for Li ions compared to that of **1**. On the other hand, the XRD patterns for **3** and **4** exhibited some sharp peaks, indicating the crystallinities of these samples (Figs S2c and S2d, respectively). Polysilanes containing two alkyl groups have been reported to exhibit strong XRD peaks at 7°–15° that are attributable to the superposition of the (110) and (020) planes of an orthorhombic lattice with hexagonally packed chains^20^.

With this structural information in hand, the anode performance of each polymer was investigated by using half cells composed of working electrodes incorporating **1**–**4**, a lithium counter electrode and 1 M LiPF_6_/EC (ethylene carbonate) + DEC (diethyl carbonate) as the electrolyte. The working electrodes were prepared by using 80% active material (**1**–**4**), 10% acetylene black and 10% carboxymethyl cellulose (CMC) as the binder. Acetylene black was added to facilitate electron transfer from the active material to the current collector. The electrode loadings ranged from 8 mg·cm^−2^ to 10 mg·cm^−2^.

**Figure 2:**
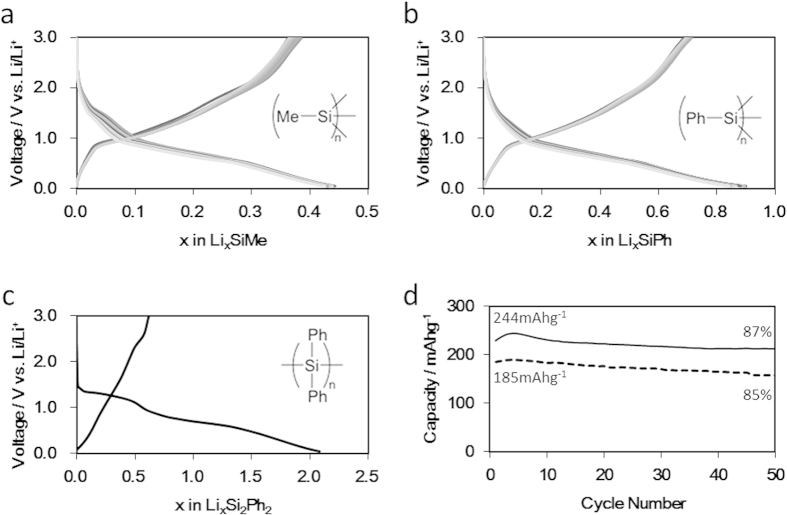
Voltage-composition profiles for polysilynes under galvanostatic cycling. Voltage profiles of (**a**) poly(methylsilyne) **1**, (**b**) poly(phenylsilyne) **2** and (**c**) poly(diphenylsilane) **4**. First 10 cycles are shown in (**a**,**b**). (**d**) Retention capacities of polysilynes **1** (solid line) and **2** (dashed line). All samples were cycled at the same charge/discharge rate of 10 mA·g^−1^. Capacities were calculated based on active materials.

**Figure 3:**
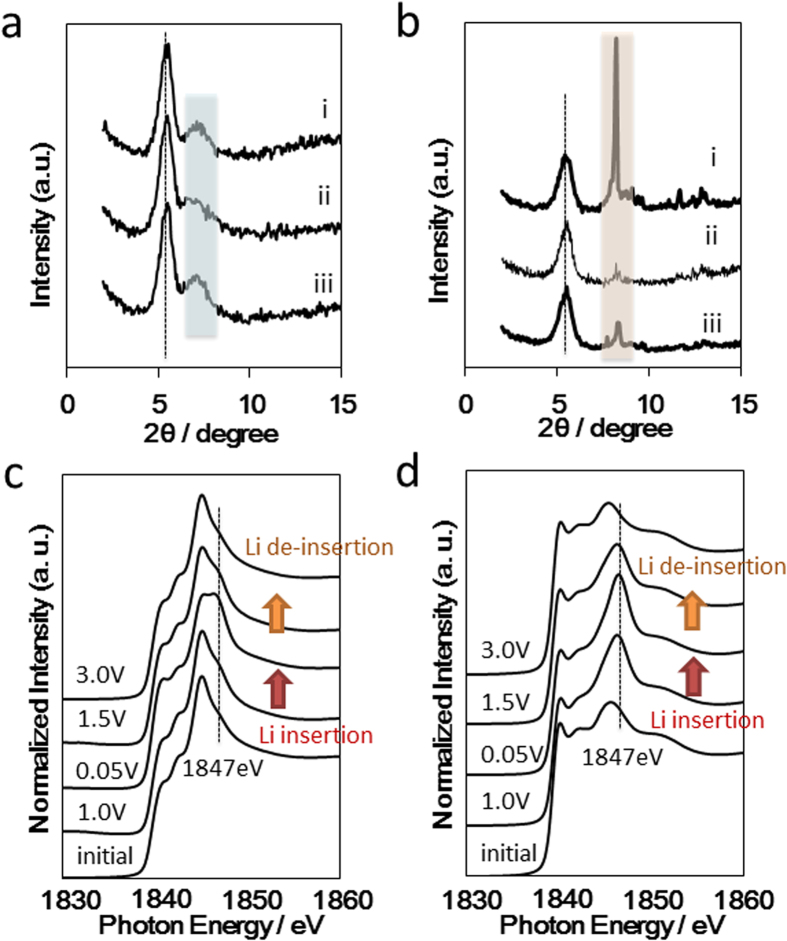
Structural data for polysilyne and polysilane during electrochemical analysis. XRD patterns for (**a**) poly(phenylsilyne) **2** and (**b**) poly(diphenylsilane) **4** at different voltages during first Li insertion and de-insertion: (i) initial, (ii) Li insertion at 0.05 V and (iii) Li de-insertion at 3.0 V. Black dotted lines represent XRD cell holder. Si K-edge XAFS spectra of (**c**) poly(methylsilyne) **1** and (**d**) poly(phenylsilyne) **2**. All spectra were obtained after adjusting each voltage to 10 mA·g^−1^.

The cells were charged and discharged within the voltage range from 0.05 V to 3.00 V at room temperature. Figure 2a,b show the first 10 cycle Voltage–composition traces for the **1**/Li and **2**/Li cells cycled at a rate of 10 mA·g^−1^. The capacities were calculated based on the active material weights in the anodes. The voltage profiles for both cells gradually change non-uniformly due to their amorphous structures. The **1**/Li cell had an overall capacity of 0.45 Li per unit formula (SiMe/Li). In contrast, the **2**/Li cell had a twice overall uptake capacity of 0.9 Li atoms per unit formula (SiPh/Li), indicating that the Li uptake capacity was affected by the organic capping group. The reason is the free volume of **2** was larger than that of **1**, as indicated by the shift of the XRD peak position for **2** to an angle lower than that of **1** (Figure S2a,b). During the following cycling, both cells had small irreversible capacities (less than 25%), likely due to the use of the carbon (acetylene black) additive, which has a large irreversible capacity (40 mAh·g^−1^ -carbon) when cycled below 1 V. For silicon and SiOC composite anodes (irreversible capacity > 100%)^21^, the irreversible capacities were extremely small and similar to that of carbon (graphite)^9^. The reversible capacities for the **1**/Li and **2**/Li cells were respectively 244 mAh·g^−1^ and 185^ ^mAh·g^−1^, and they retained 87% and 85%, respectively, of their initial capacities after 50 cycles (Fig. 2d). These capacities were calculated by dividing by the molecular weights of the active materials, and therefore the capacity for **1** capped with a Me moiety was larger than that for **2** capped with a Ph moiety. Although the general reason for such fading is considered to be a structural change or the solubility of the electrolyte, no discoloration was observed upon dismantling of the cell. It is thought that the 110% change in the volume of the polysilynes upon insertion and extraction of lithium (Fig. 3a) results in pulverization and capacity fading. Furthermore, **3/**Li and **4/**Li, which have Si–Si chain structures, exhibited large irreversibilities (>70%) (Fig. 2c for **4**/Li). These features imply that polymers **1** and **2** are more likely to yield anodes with cycle stability than are the chain-structured silicon polymers **3** and **4**.

### Mechanisms of redox properties

These results suggest that there are interesting differences between the polysilynes (**1**, **2**) and polysilanes (**3**, **4**) that are related to their electrochemical reversibilities. Therefore, the Li insertion/de-insertion processes for polysilyne **2** and polysilane **4** were monitored by using XRD (Fig. 3a,b, respectively). After Li insertion at 0.05 V, the XRD pattern of **2** exhibits a very slight low-angle shift of the broad signal observed in the starting sample (Fig. 3a, blue portion). This small shift indicates that the polysilyne **2** unit cell increased due to Li adsorption in the network (Fig. 3a). After Li de-insertion at 3.0 V, the XRD pattern is very similar to that of the starting sample, indicating excellent reversibility of Li ion insertion in **2**. In the case of **4**, the sharp peaks observed in the XRD pattern of the starting sample disappear after Li insertion and do not reappear following de-insertion (Fig. 3b). This result indicates that the Si–Si bonds of **4** were attacked by the Li ions and then collapsed, which agrees well with the reversibility behaviour observed in the electrochemical tests (Fig. 2c). Thus, for polysilyne **2**, the Si–Si network was mainly retained during electrochemical cycling, whereas for polysilane **4**, the Si–Si chain structure was broken during Li insertion.

In addition to the XRD analyses, the local environments of the silicon frameworks of the polysilynes were examined by Si K-edge X-ray absorption near-edge structure (XANES) analysis. Polysilynes **1** and **2** exhibited moderate spectral shapes originating at 1839 eV with peaks at 1841 eV, 1842 eV and 1845 eV (Fig. 3c,d, respectively). The peak observed at 1841 eV has the same energy as that for the Si–Si bond in crystalline silicon^22^, while the peaks observed at 1842 eV and 1845 eV correspond to those of Si–C bonds in SiC wafers^23^. After Li insertion, a new peak appeared at approximately 1847 eV, and its intensity increased as Li insertion progressed. The simulated XANES spectra of Si-Li structural models for layered polysilane (Si_6_H_6_) based on the FEFF program also included a new high-energy peak (1850 eV)^24^. It should also be noted that the intensity ratio of the peaks of **1** and **2** at 1847 eV was approximately 1:2, which is in good agreement with the electrochemically measured Li insertion capacities. It was thus concluded that the redox centres in the Si–Si bonds of the polysilynes were partially replaced with Si–Li bonds after Li insertion. During Li de-insertion, the peak at 1847 eV gradually disappeared, and the final XANES pattern was very similar to that of the starting sample, which is also in good agreement with the electrochemical reversibility results. It should be noted that the three-dimensional networks of polysilynes **1** and **2** are important in the electrochemical performance, the Si–Li bonds in reduced polysilyne **1** and **2** can be changed into Si–Si bonds. On the other hand, the chain structures of polysilanes **3** and **4** collapsed after formation of the Si–Li bonds. As a result, the Si–Si bonds in **3** and **4** could not be reformed. Therefore, the chain structures of these polysilanes could not be reconstructed because of the very long distance between the Si atoms. In addition, the cycle stabilities of polysilynes **1** and **2** were nearly the same, which indicates that the organic capping moieties did not affect their electrochemical stabilities. And also, we previously reported that Si–phenyl bond of phenyl capped lithiosilane was stable, in which the two lithium atoms are associated with the two phenyl groups^25^. Based on all of the present results and previous report, it was thus concluded that the stabilities of polysilynes **1** and **2** were enhanced due to the introduction of the capping groups.

### Lithiation of the polysilynes via a mechanochemical reaction

As mentioned above, it was demonstrated that Li ions were electrochemically inserted into the polysilynes. However, Li-ion absorption into polysilynes **1** and **2** (polysilyne/nLi, n = 0.5, 1.0) also occurred via a mechanochemical solid phase reaction. In a typical synthesis, polysilyne **1** (100 mg, 2.33 mmol of the SiMe unit) or **2** (100 mg, 0.95 mmol of the SiPh unit) and Li (1:8.08 mg for n = 0.5 and 1:16.2 mg for n = 1.0 or 2:3.31 mg for n = 0.5 and 2:6.62 mg for n = 1.0) were placed in a mortar and ground using a pestle at room temperature under an argon atmosphere. After milling, the Li fragments completely disappeared, and the yellow powdered polysilyne changed into a black powder. To investigate the changes in the electronic band structure, solid-state diffuse reflectance UV-vis spectra were recorded.

**Figure 4:**
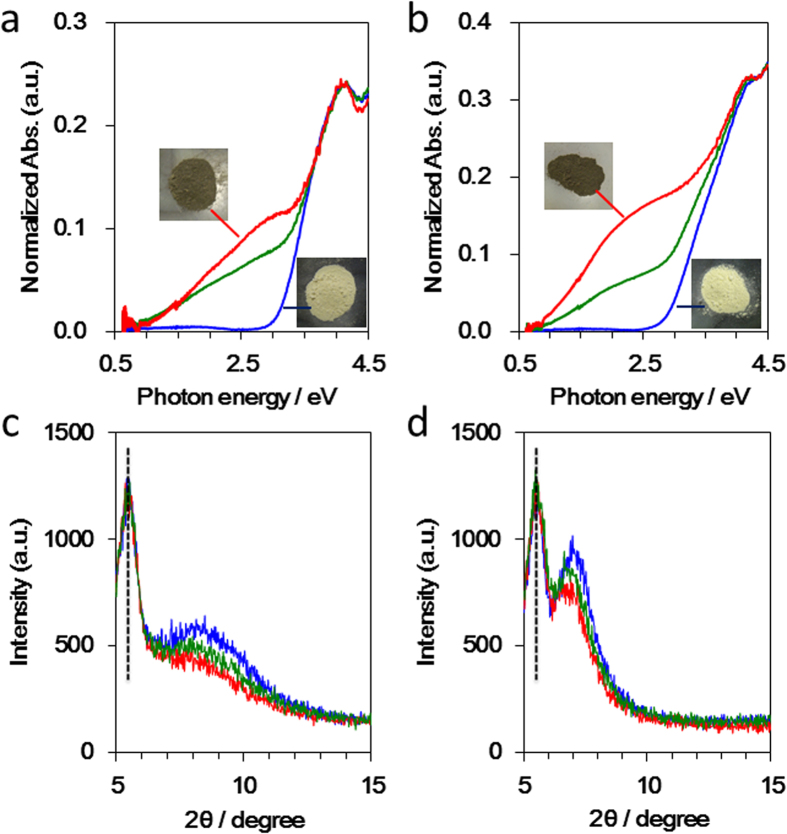
Structural evolution of polysilynes during lithiation. Diffuse reflectance UV-vis spectra of (**a**) poly(methylsilyne) **1** and (**b**) poly(phenylsilyne) **2**. Corresponding photos for each sample are also shown (insets). Colour change with lithiation was observed. XRD patterns of (**c**) poly(methylsilyne) **1** and (**d**) poly(phenylsilyne) **2**. Blue, green and red lines are initial pattern and patterns for SiR/nLi (n = 0.5) and SiR/nLi (n = 1.0), respectively. Black dotted lines represent XRD cell holder.

Poly(methylsilyne) **1** exhibited a strong absorption band in the UV region with an edge at 450 nm (3.0 eV), which is in agreement with that of the previously reported Si polymer (Fig. 4a). After the mechanochemical reaction, the absorption band edge of polysilyne **1**/0.5 Li shifted to a longer wavelength and exhibited a lower bandgap energy (0.8 eV). Increasing the Li content to yield polysilyne **1**/1.0 Li did not result in a further shift of this absorption peak, suggesting that the Li storage capacity of **1** is saturated above a composition of **1**/0.5 Li. Poly(phenylsilyne) **2** exhibited an absorption band edge at 490 nm (2.8 eV) very similar to that of poly(methylsilyne) **1** (Fig. 4b). After milling **2** with 0.5 Li, both polysilyne **2** and polysilyne **2**/0.5 Li-like moieties coexisted in the composite due to the inhomogeneous distribution of Li in polysilyne **2**/0.5 Li. However, when an equal amount of Li was added to generate polysilyne **2**/1.0 Li, the peak shape drastically changed and exhibited an absorption edge at 0.8 eV. In addition, in the XRD patterns shown in Fig. 4c,d, it can be seen that the peak shift for n = 0.5 for polysilyne **1**/n Li is saturated at 2θ = 8.0°, while the peak for polysilyne **2** shifts continuously to 2θ = 6.6° from 7.2° as the amount of added Li increases. These results agree well with those obtained from the electrochemical Li insertion reaction analysis, for which values of 0.45 Li per formula unit and 0.9 Li per formula unit of polysilyne **1** and polysilyne **2**, respectively, were determined. In other words, the quantity of Li that could be inserted increased when the polysilyne included an organic substituent that expanded the cage size. Furthermore, these results indicate the formation of new electron band levels following lithiation of the polysilynes. This property renders the polysilynes suitable for use as anode materials, because their conductivities increase during the Li insertion process.

### Electrode morphology at low temperature

**Figure 5:**
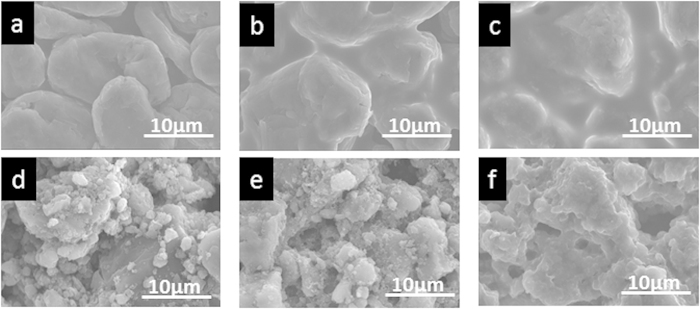
SEM images of different anodes before and after Li insertion. Initial surface of anodes based on (**a**) graphite and (**d**) poly(methylsilyne) **1**. SEM images of (**b**,**c**) graphite and (**e**,**f**) poly(methylsilyne) **1** after Li insertion at 10 mA·g^−1^ and 100 mA·g^−1^, respectively.

Finally, to assess the low-temperature performances of these new anode materials, cells were prepared in which (i) commercial graphite (Fig. 5a–c) or (ii) polysilyne **1** (Fig. 5d–f) was paired with Li metal in a half-cell operated at 5 °C. These cells were subjected to deep Li insertion to 0.05 V and then extracted for SEM observation. After the first Li insertion at 10 mA·g^−1^, the graphite electrode was covered with thin mossy Li compounds or a solid electrolyte interface (Fig. 5b). Furthermore, when the applied current density was increased to 100 mA·g^−1^, the mossy layer grew very thick (Fig. 5c) and individual graphite particles could not be clearly observed in the anode. The situation was very different with polysilyne **1**. After the first Li insertion at a rate of 10 mA·g^−1^, neither mossy Li compounds nor a solid electrolyte interface was clearly observed on the electrode surface (Fig. 5e), similarly to that of the initial polysilyne **1** (Fig. 5d). Even after applying a current density of 100 mA·g^−1^, individual particles of polysilyne **1** could still be observed, although very thin mossy Li compounds covered the electrode. The lack of Li compound formation on the polysilyne electrode is attributed to the moderate Li insertion potential of approximately 1 V. Due to the polymeric silicon structure of polysilyne **1**, the electrolyte wetted both the outside and inside surfaces of the anode, which had a highly lipophilic character. Thus, it is thought that the mobility of the Li ions in the polysilyne anode was higher than that in the graphite anode. In addition, the potential for Li insertion is very close to that of Li metal formation for the graphite anode, and therefore, Li compounds readily formed on the electrode surface. The stability of the anode material surface is known to be important for battery cycling and safety. Thus, the present results suggest that polysilyne is a suitable anode material for LIBs operated at low temperatures or high rates due to its stability.

## Conclusions

Polysilynes were proposed as anode materials that can successfully address the safety concerns associated with the anode materials currently used in LIBs. The results obtained for the polysilynes may lead to the development of new types of anode materials containing Si frameworks that can safely be applied in the next generation of EV batteries. Future studies are planned to further develop the design of the Si framework and to optimize the organic substituents in order to increase their performances.

## Methods

### Synthesis of 1–4

**1**: In an argon atmosphere, a solution containing methyltrichlorosilane (2.17 g, 14.5 mmol) and hexamethyl phosphoramide (HMPA, 3 ml) was added dropwise within 5 min (with high-speed stirring) to a sodium dispersion (0.97 g, 42.0 mmol) in refluxing toluene (40 ml). The mixture was stirred and refluxed for 9 h and then treated with MeLi (2.2 ml of a 1.1 M solution in diethyl ether) at 50 °C for 2 h, followed by cooling. The excess sodium was quenched with MeI, and the resulting mixture was washed with water, hexane and toluene to remove salts and excess monomers. After filtering the mixture, **1** was obtained as an insoluble pale yellow powder (0.57 g, 92% Si yield based on the starting materials).

**2**: In an argon atmosphere, a solution containing phenyltrichlorosilane (3.16 g, 14.9 mmol) and HMPA (3 ml) was added dropwise within 5 min (with high-speed stirring) to a sodium dispersion (1.00 g, 43.3 mmol) in refluxing toluene (40 ml). The mixture was stirred and refluxed for 9 h and then treated with MeLi (2.2 ml of a 1.1 M solution in diethyl ether) at 50 °C for 2 h, followed by cooling. The excess sodium was quenched with MeI, and the resulting mixture was washed with water, hexane and toluene to remove salts and excess monomers. After filtering, **2** was obtained as an insoluble yellow powder (1.06 g, 68% Si yield based on the starting materials).

**3**: In an argon atmosphere, a solution containing dimethyldichlorosilane (5.90 g, 45.7 mmol) and HMPA (6 ml) was added dropwise within 5 min (with high-speed stirring) to a sodium dispersion 2.10 g (91.3 mmol) in refluxing toluene (80 ml). The mixture was stirred and refluxed for 9 h and then treated with MeLi (7.0 ml of a 1.1 M solution in diethyl ether) at 50 °C for 2 h, followed by cooling. The excess sodium was quenched with MeI, and the resulting mixture was washed with water, hexane and toluene to remove salts and excess monomers. After filtering, **3** was obtained as an insoluble white powder (0.56 g, 21% Si yield based on the starting materials).

**4**: In an argon atmosphere, a solution containing diphenyldichlorosilane (11.6 g, 45.7 mmol) and HMPA (6 ml) was added dropwise within 5 min (with high-speed stirring) to a sodium dispersion 2.10 g (91.3 mmol) in refluxing toluene (80 ml). The mixture was stirred and refluxed for 9 h and then treated with MeLi (7.0 ml of a 1.1 M solution in diethyl ether) at 50 °C for 2 h, followed by cooling. The excess sodium was quenched with MeI, and the resulting mixture was washed with water, hexane and toluene to remove salts and excess monomers. After filtering, **4** was obtained as an insoluble white powder (4.90 g, 59% Si yield based on the starting materials).

### Electrochemical Tests

The electrodes were prepared by separately mixing **1**–**4** and acetylene black (AB) with CMC as a binder (ratio: 8/1/1 wt%). The materials were mixed by using water as a solvent, and the resulting paste was coated on a copper sheet by using a coater. Next, water was removed under a vacuum at 100 °C for 16 h. Hermetically sealed two-electrode cells were used for the electrochemical experiments. The working electrode (0.5 mg) was separated from the lithium anode by a porous polyethylene film imbibed with 1 M LiPF_6_/EC+DEC. The three layers were pressed between two current collectors, one in contact with the active material, and the other in contact with a lithium disk. CV experiments and charge–discharge tests were performed by using HZ5000 and HJ1010 mSM8A instruments (Hokuto), respectively. All of the tests were performed within the voltage range from 0.05 V to 3.00 V at 27 °C, and the CV measurements were performed at a sweep rate of 0.3 mV·s^−1^. The current density applied for the charge–discharge tests was 25 mA·g^−1^. The capacities were determined based on the weights of **1**–**4** in the electrodes.

### Characterization

The XRD patterns were obtained by using a RintTTR instrument (Rigaku, Japan). The analyses were conducted under an argon atmosphere at room temperature by using CuKα radiation (50 KV, 300 mA).

The Si K-edge XANES analyses were performed on the BL-10 double crystal monochromator beamline by using an InSb(111) crystal pair at the SR Centre of Ritsumeikan University. The spectra were obtained by using the total electron yield mode.

### Mechanical Milling

The SiR/nLi composites were prepared via a mechanochemical solid-phase reaction. Poly(methylsilyne) **1** (100 mg, 2.33 mmol of the SiMe unit) and Li (8.08 mg for n = 0.5 and 16.2 mg for n = 1) were placed in a mortar and were milled by using a pestle at room temperature under an argon atmosphere. Alternatively, poly(phenylsilyne) **2** (100 mg, 0.95 mmol of the SiPh unit) and Li (3.31 mg for n = 0.5 and 6.61 mg for n = 1) were milled.

### Solid-State Diffuse Reflectance UV-Vis Analysis

The solid-state diffuse reflectance UV-vis spectra were recorded on a JASCO V-670 spectrophotometer with an integrating sphere unit (JASCO ISN-723). The samples were encapsulated in a sealed cell under an argon atmosphere.

## Additional Information

**How to cite this article**: Maruyama, H. *et al.* Improving battery safety by reducing the formation of Li dendrites with the use of amorphous silicon polymer anodes. *Sci. Rep.* **5**, 13219; doi: 10.1038/srep13219 (2015).

## Supplementary Material

Supplementary Information
