# Supplementary material for: Improving battery safety by reducing the formation of Li dendrites with the use of amorphous silicon polymer anodes
Source: Sci Rep. 2015 Aug 7;5:13219. doi: 10.1038/srep13219 (PMC4528197; doi:10.1038/srep13219)
Supplement: Supplementary Information [file srep13219-s1.pdf]

Supplementary information

Improving battery safety by reducing the formation of Li dendrites with the use of amorphous silicon polymer anodes

Hitoshi Maruyama[1], Hideyuki Nakano[2], Masahiro Ogawa[3], Masaaki Nakamoto[1], Toshiaki Ohta[3], Akira Sekiguchi[1]

[1]Department of Chemistry, Graduate School of Pure and Applied Sciences, University of Tsukuba, Tsukuba, Ibaraki 305-8571, Japan

[2]TOYOTA CENTRAL R&D LABS., INC., Nagakute, Aichi 480-1192, Japan

[3]SR Center, Ritsumeikan University, 1-1-1 Noji-Higashi, Kusatsu, Shiga, 525-8577, Japan

To whom correspondence should be addressed. E-mail: hnakano@mosk.tytlabs.co.jp and sekiguch@chem.tsukuba.ac.jp

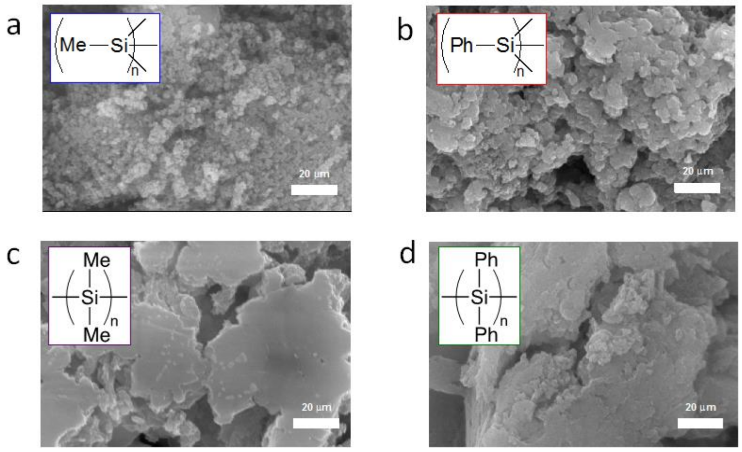

Figure S1 │ Scanning electron microscope images.

a, poly(methylsilyne) **1**, **b**, poly(phenylsilyne) **2**, **c**, poly(dimethylsilane) **3**, and **d**, poly(diphenylsilane) **4**.

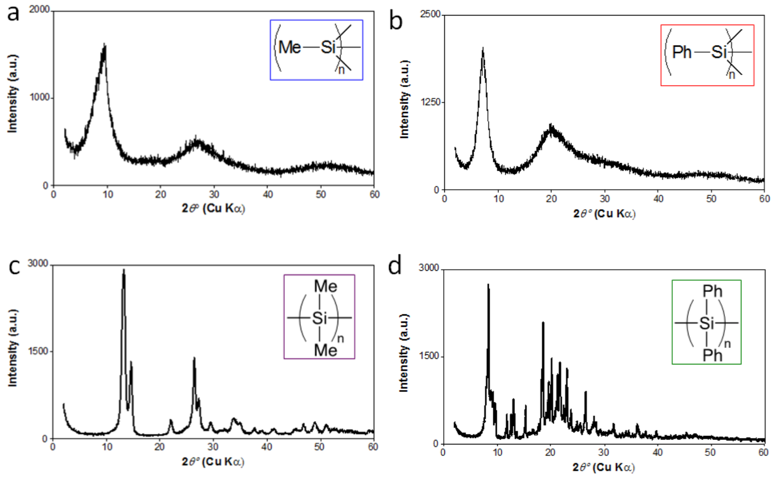

Figure S2 │ XRD patterns. a, poly(methylsilyne) **1**, **b**, poly(phenylsilyne) **2**, **c**, poly(dimethylsilane) **3**, and **d**, poly(diphenylsilane) **4**.
